# Supplementary material for: The world seems different in a social context: A neural network analysis of human experimental data
Source: PLoS One. 2022 Aug 30;17(8):e0273643. doi: 10.1371/journal.pone.0273643 (PMC9426934; doi:10.1371/journal.pone.0273643)
Supplement: S1 Appendix — See Appendix for detailed description of the effect of Bayesian inference on the integration of sensory and prior information. (PDF) [file pone.0273643.s001.pdf]

Appendix: Effect of Bayesian inference on the integration of sensory and prior information

To demonstrate that nonlinear changes in figures 5a and 6a from the main manuscript are caused by the mathematical formulation of Bayesian inference as shown in equations 2 and 3 in the main manuscript, we calculated the result of the Bayesian inference using a simple example.

The pseudocode for this calculation is provided as Algorithm 1. Specifically, as an example the input of the current time step is assumed to be 1, with a sensory variance of $\sigma^2_{\text{sensor}} = 0.001$ and the expected value (i.e. the network's prior) is 0.5 with variance of $\sigma^2_{\text{sensor}} = 0.01$. By either fixing H_{sensor} while modifying H_{prior} (lines 8-14) or fixing H_{prior} while modifying H_{sensor} (lines 15-20), the result of the Bayesian inference under different H values can be computed.

Algorithm 1 Pseudocode for performing Bayesian inference with different ratios of H_{prior} and H_{sensor}

1: **procedure** SimpleBayesianInferenceExample
2: input $\leftarrow 1$
3: $\sigma^2_{\text{sensor}} \leftarrow 0.001$
4: $\mu_{\text{prior}} \leftarrow 0.5$
5: $\sigma^2_{\text{prior}} \leftarrow 0.01$
6: priorList $\leftarrow [1, 0.5, 0.45, 0.4, 0.35, 0.3, 0.25, 0.2, 0.15, 0.1, 0.09, 0.08, 0.07, 0.06, 0.05]$
7: sensorList $\leftarrow [\frac{1}{z} \forall z \in \text{priorList}]$
8: $\text{H}_{\text{sensor}} \leftarrow 1$
9: **for** i in length(priorList) **do**
10: $\text{H}_{\text{prior}} \leftarrow \text{priorList}[i]$
11: $\sigma^2_{\text{post}} = \frac{(\sigma^2_{\text{sensor}} \cdot H_{\text{sensor}}) \cdot (\sigma^2_{\text{prior}} \cdot H_{\text{prior}})}{(\sigma^2_{\text{sensor}} \cdot H_{\text{sensor}}) + (\sigma^2_{\text{prior}} \cdot H_{\text{prior}})}$
12: $\mu_{\text{post}} = \sigma^2_{\text{post}} \cdot \left(\frac{\mu_{\text{prior}}}{\sigma^2_{\text{prior}} \cdot H_{\text{prior}}} + \frac{\text{input}}{\sigma^2_{\text{sensor}} \cdot H_{\text{sensor}}} \right)$
13: $\text{posterior}[i] \leftarrow \mu_{\text{post}}$
14: plot (x,y) where $x \in$ priorList, $y \in$ posterior ($\rightarrow$ Fig. 1a)
15: $\text{H}_{\text{prior}} \leftarrow 1$
16: **for** i in length(sensorList) **do**
17: $\text{H}_{\text{sensor}} \leftarrow \text{priorList}[i]$
18: $\sigma^2_{\text{post}} = \frac{(\sigma^2_{\text{sensor}} \cdot H_{\text{sensor}}) \cdot (\sigma^2_{\text{prior}} \cdot H_{\text{prior}})}{(\sigma^2_{\text{sensor}} \cdot H_{\text{sensor}}) + (\sigma^2_{\text{prior}} \cdot H_{\text{prior}})}$
19: $\mu_{\text{post}} = \sigma^2_{\text{post}} \cdot \left(\frac{\mu_{\text{prior}}}{\sigma^2_{\text{prior}} \cdot H_{\text{prior}}} + \frac{\text{input}}{\sigma^2_{\text{sensor}} \cdot H_{\text{sensor}}} \right)$
20: $\text{posterior}[i] \leftarrow \mu_{\text{post}}$
21: plot (x,y) where $x \in$ sensorList, $y \in$ posterior ($\rightarrow$ Fig. 1b)

Fig. 1 plots the posterior mean resulting from these calculations. It can be seen that the curve in Fig. 1a follows a similar trend as in Fig. **??**. Similarly, Fig. 1b shows a decreasing posterior mean with an increasing value of H_{sensor}

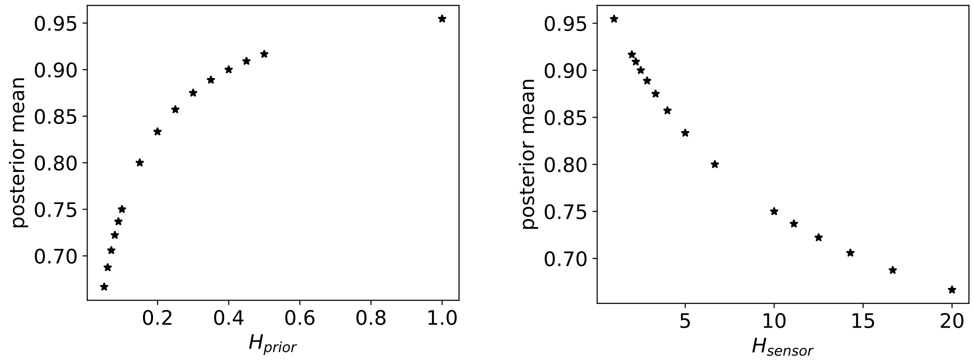

(a) Fixing $H_{\text{sensor}} = 1$ **(b)** Fixing $H_{\text{prior}} = 1$

Fig. 1. Results computed by Algorithm 1: the posterior mean resulting from the Bayesian inference when (a) keeping H_{sensor} fixed while modifying H_{prior}, and (b) when keeping H_{prior} fixed while modifying H_{sensor}.
